# Supplementary material for: Risk Factors and Ocular Health Associated with Toxoplasmosis in Quilombola Communities
Source: Microorganisms. 2026 Jan 1;14(1):96. doi: 10.3390/microorganisms14010096 (PMC12844210; doi:10.3390/microorganisms14010096)
Supplement: Supplementary file 1 [file microorganisms-14-00096-s001.zip › microorganisms-3986349-supplementary.pdf]

Table S1. Sociodemographic characteristics, analysis of epidemiological and ocular characteristics related to *Toxoplasma gondii* seropositivity in the *quilombola* children.

Sociodemographic, epidemiological and ocular health factors		n (Positive)/ Total		n (Negative)/ Total		OR	CI95%	*P*
		n.	%	n.	%			
Active chorioretinitis presence	Yes	0	0.00	0	0.00	-	-	-
	No	38	100.00	43	100.00			
Anterior chamber and iris	Altered	0	0.00	0	0.00	-	-	-
	Normal	38	100.00	43	100.00			
Astigmatism	Yes	9	23.68	11	25.58	0.90	0.33-2.49	0.843
	No	29	76.32	32	74.42			
Bathroom with toilet bowl	Yes	34	89.47	40	93.02	0.64	0.13-3.05	0.570
	No	4	10.53	3	6.98			
Chorioretinitis scar lesion	Yes	0	0.00	0	0.00	-	-	-
	No	38	100.00	43	100.00			
Cornea	Altered	0	0.00	0	0.00	-	-	-
	Normal	38	100.00	43	100.00			
Distance visual acuity (DVA)	20/20	29	76.32	30	69.77	-	-	-
	20/30 - 20/60	7	18.42	11	25.58	0.66	0.22-1.91	0.621
	20/70 - 20/160	1	2.63	1	2.33	1.03	0.07-18.79	0.487
	20/200 - 20/400	0	0.00	1	2.33	-	-	0.344
	Blindness (CD)	0	0.00	0	0.00	-	-	-
	SPL	0	0.00	0	0.00	-	-	-
	PL	0	0.00	0	0.00	-	-	-
Drinks water from a well, pond, or river	Yes	13	34.21	8	18.60	2.27	0.82-6.30	0.356
	No	25	65.79	35	81.40			
Eats raw or undercooked meat	Yes	8	21.05	6	13.95	1.64	0.51-5.26	0.399
	No	30	78.95	37	86.05			
Eye deviation	Yes	0	0.00	0	0.00	-	-	-
	No	38	100.00	43	100.00			
Goes to a farm, ranch, or poultry farm	Yes	29	76.32	34	79.07	0.85	0.30-2.43	0.766
	No	9	23.68	9	20.93			
Goes to the beach	Yes	19	50.00	18	41.86	1.39	0.58-3.34	0.463
	No	19	50.00	25	58.14			
Has a Garden	Yes	25	65.79	15	34.88	**3.59**	**1.43-8.99**	**0.005**

	No	13	34.21	28	65.12			
Has a yard with soil	Yes	38	100.00	42	97.67	-	-	0.344
	No	0	0.00	1	2.33			
Has any illness	Yes	1	2.63	0	0.00	-	-	0.284
	No	37	97.37	43	100.0			
Has cats	Yes	23	60.53	10	23.25	**5.06**	**1.93-13.23**	**0.001**
	No	15	39.47	33	76.74			
Has dogs	Yes	31	81.58	29	67.44	2.14	0.76-6.04	0.147
	No	7	18.42	14	32.56			
Has had parasitic infections.	Yes	21	55.26	23	53.49	1.07	0.45-2.58	0.873
	No	17	44.74	20	46.51			
Has noticed any vision problems	Yes	3	7.89	6	13.95	0.53	0.12-2.28	0.386
	No	35	92.11	37	86.05			
Household waste disposal	Septic tank	34	89.47	41	95.35	0.41	0.07-2.40	0.314
	Open air	4	10.53	2	4.65			
Lens	Opaque	0	0.00	0	0.00	-	-	-
	Normal	38	100.00	43	100.00			
Maternal ethnicity	mixed race	20	52.63	23	54.49	0.97	040-2.32	0.88
	Black	18	47.37	20	46.51			
Ocular itching	Yes	0	0.00	0	0.00	-	-	-
	No	38	100.00	43	100.00			
Ocular motility	Normal	38	100.00	43	100.00	-	-	-
	Permanent exotropia (XT)	0	0.00	0	0.00			
Ocular redness	Yes	0	0.00	0	0.00	-	-	-
	No	38	100.00	43	100.00			
Other illnesses	Arthritis	0	0.00	0	0.00	-	-	0.93
	Depression	0	0.00	0	0.00	-	-	0.876
	Diabetes	0	0.00	0	0.00	-	-	0.932
	Dyslipidemia	0	0.00	0	0.00	-	-	0.550
	Gastritis	0	0.00	0	0.00	-	-	0.345
	Arterial hypertension	0	0.00	0	0.00	-	-	0.094
	Renal insufficiency	0	0.00	0	0.00	-	-	0.345
Parents' education level	≤ 8 years	19	50.00	17	39.53	0.65	0.27-1.58	0.344
	> 8 years	19	50.00	26	60.47			

Participant's or guardian's education level (for children)	≤ 8 years	38	100.0	42	97.67	-	-	0.344
	> 8 years	0	0.00	1	2.33			
Plays in the soil	Yes	35	92.11	21	95.35	**12.22**	**3.26-45.85**	**0.0001**
	No	3	7.89	22	4.65			
Pupil	Normal	38	100.00	43	100.00	-	-	-
	Relative Afferent Pupillary Defect (RAPD)	0	0.00	0	0.00	-	-	-
	Paralytic mydriasis	0	0.00	0	0.00	-	-	-
	Paralyzed at mid-dilation	0	0.00	0	0.00	-	-	-
Reduced visual acuity (RVA)	Yes	0	0.00	0	0.00	-	-	-
	No	38	100.00	43	100.00			
Regular garbage collection	Yes	25	65.79	27	62.79	1.14	0.46-2.84	0.778
	No	13	34.21	16	37.21			
Sex	Female	19	50.00	20	46.51	1.15	0.48-2.75	0.755
	Male	19	50.00	23	53.49			
Source of water for the garden	Treated water	9	23.68	7	16.28	1.60	0.53-4.80	0.403
	Stream/River	3	7.89	0	0.00	-	-	0.060
	Rain	0	0.00	0	0.00	-	-	0.216
	Well/Reservoir	13	34.21	8	18.60	2.27	0.82-6.30	0.110
Spherical equivalente	Normal vision	28	73.68	32	74.42	-	-	-
	Myopia	8	21.05	6	13.95	1.52	0.47-4.93	0.682
	Hyperopia	2	5.26	5	11.63	0.47	0.08-2.54	0.610
Swims in the river, lagoon, or pond	Yes	3	7.89	0	0.00	-	-	0.060
	No	35	92.11	43	100.00			
Vascular narrowing	Present	0	0.00	0	0.00	-	-	-
	Present	38	100.00	43	100.00			
Vasculitis	Present	0	0.00	0	0.00	-	-	-
	Absent	38	100.00	43	100.00			
Vitritis	Present	0	0.00	0	0.00	-	-	-
	Absent	38	100.00	43	100.00			
There are cats in the neighborhood	Yes	30	78.95	30	69.77	1.62	0.59-4.49	0.347
	No	8	21.05	13	30.23			
There are dogs in the neighborhood	Yes	30	81.08	37	90.24	0.46	0.12-1.73	0.246
	No	7	18.92	4	9.76			

Walks barefoot	Yes	28	73.68	29	67.44	1.35	0.52-3.54	0.539
	No	10	26.32	14	32.56			
Water source	Piped	22	57.89	29	67.44	1.51	0.61-3.73	0.374
	Well	16	42.11	14	32.56			
Which animal(s) do you have?	None	11	28.95	11	25.58	1.18	0.44-3.16	0.734
	Horse	9	23.68	6	13.95	1.91	0.61-5.99	0.26
	Rabbit	2	5.26	0	0.00	-	-	0.128
	Chicken	22	57.89	29	67.44	0.66	0.27-1.64	0.374
	Bird	2	5.26	2	4.65	1.14	0.15-8.50	0.899
	Pig	5	13.16	4	9.30	1.48	0.37-5.95	0.375
	Pigeon	0	0.00	0	0.00	-	-	-
	Cow	7	18.42	8	18.60	0.99	0.32-3.04	0.983

n: number, %: percentage, OR: Odds Ratio, CI: Confidence Interval, *p*: Significance Level

Table S2. Sociodemographic characteristics, analysis of epidemiological and ocular characteristics related to *Toxoplasma gondii* seropositivity in elderly *quilombolas*

Sociodemographic, epidemiological and ocular health factors		n (Positive)/ Total		n (Negative)/ Total		OR	CI95%	*P*
		n.	%	n.	%			
Active chorioretinitis presence	Yes	0	0.00	62	100.00	-	-	-
	No	0	0.00	18	100.00			
Anterior chamber and íris	Altered	0	0.00	0	0.00	-	-	-
	Normal	62	100.0	18	100.00			
Astigmatism	Yes	28	45.16	10	55.56	0.66	0.23-1.89	0.437
	No	34	54.84	8	44.44			
Bathroom with toilet bowl	Yes	59	95.16	18	100.0	-	-	0.341
	No	3	4.84	0	0.00			
Chorioretinitis scar lesion	Yes	3	5.00	1	5.56	1.12	0.11-11.45	0.925
	No	57	95.00	17	94.44			
Cornea	Altered	5	8.20	0	0.00	-	-	0.209
	Normal	56	91.80	18	100.00			
Distance visual acuity (DVA)	20/20	5	8.06	1	5.56	-	-	-
	20/30 - 20/60	14	22.58	8	44.44	0.35	0.03-3.55	0.672
	20/70 - 20/160	19	30.65	6	33.33	0.63	0.06-6.54	0.875
	20/200 - 20/400	8	12.90	1	5.56	1.60	0.29-21.60	0.642
	Blindness (CD)	12	19.35	1	5.56	2.40	0.49-33.75	0.834
	SPL	4	6.45	0	0.00	-	-	0.269
	PL	0	0.00	1	1.64	-	-	0.062
Drinks water from a well, pond, or river	Yes	8	12.90	1	5.56	2.52	0.29-21.60	0.385
	No	54	87.10	17	94.44			
Eats raw or undercooked meat	Yes	10	16.13	5	27.78	0.50	0.14-1.72	0.265
	No	52	83.87	13	72.22			
Eye deviation	Yes	1	1.61	0	0.00	-	-	0.588
	No	61	98.39	18	100.00			
Goes to a farm, ranch, or poultry farm	Yes	22	35.48	7	38.89	0.86	0.29-2.55	0.791
	No	40	64.52	11	61.11			
Goes to the beach	Yes	15	24.19	5	27.78	0.83	0.25-2.71	0.757
	No	47	75.81	13	72.22			
Has a Garden	Yes	40	64.52	5	50.00	**4.73**	**1.49-15.00**	**0.013**

	No	22	35.48	13	50.00			
Has a yard with soil	Yes	**57**	**91.94**	**11**	**61.11**	**7.25**	**1.94-27.08**	**0.004**
	No	**5**	**8.06**	**7**	**38.89**			
Has any illness	Yes	37	59.68	10	55.56	1.18	0.41-3.41	0.754
	No	25	40.32	8	44.44			
Has cats	Yes	40	64.52	5	27.78	**4.72**	**1.49-15.01**	**0.013**
	No	22	35.48	13	72.22			
Has dogs	Yes	27	43.55	12	66.67	0.39	0.13-1.16	0.084
	No	35	56.45	6	33.33			
Has had parasitic infections.	Yes	47	75.81	11	61.11	1.99	0.66-6.06	0.219
	No	15	24.19	7	38.89			
Has noticed any vision problems	Yes	60	96.77	17	94.44	1.76	0.15-20.66	0.647
	No	2	3.23	1	5.56			
Household waste disposal	Septic tank	56	90.32	18	100.00	-	-	0.170
	Open air	6	9.68	0	0.00			
Lens	Opaque	37	67.27	10	58.82	1.44	0.47-4.40	0.522
	Normal	18	32.73	7	41.18			
Maternal ethnicity	mixed race	19	30.65	6	33.33	0.88	0.29-2.70	0.942
	Black	43	69.35	12	66.67			
Ocular itching	Yes	7	11.29	2	11.11	1.02	0.19-5.39	0.983
	No	55	88.71	16	88.89			
Ocular motility	Normal	61	98.39	18	100.00	-	-	0.588
	Permanent exotropia (XT)	1	1.61	0	0.00			
Ocular redness	Yes	8	12.90	3	16.67	0.74	0.17-3.14	0.683
	No	54	87.10	15	83.33			
Other illnesses	Arthritis	2	3.23	0	0.00	-	-	0.440
	Depression	2	3.23	0	0.00	-	-	0.543
	Diabetes	11	17.74	4	22.22	0.75	0.21-2.74	0.66
	Dyslipidemia	8	12.90	4	22.22	0.52	0.14-1.97	0.330
	Gastritis	3	4.84	0	0.00	-	-	0.341
	Arterial hypertension	24	38.71	11	61.11	0.40	0.14-1.18	0.092
	Renal insufficiency	3	4.84	0	0.00	-	-	0.341
Parents' education level	≤ 8 years	61	98.39	18	100.0	-	-	0.588
	> 8 years	1	1.61	0	0.00			

Participant's or guardian's education level (for children)	≤ 8 years	52	83.87	15	83.33	0.96	0.23-3.95	0.956
	> 8 years	10	16.13	3	16.67			
Plays in the soil	Yes	33	53.23	7	38.89	1.79	0.61-5.22	0.284
	No	29	46.77	11	61.11			
Pupil	Normal	58	93.55	18	100.00	-	-	-
	Relative Afferent Pupillary Defect (RAPD)	2	3.23	0	0.00	-	-	-
	Paralytic mydriasis	1	1.61	0	0.00	-	-	-
	Paralyzed at mid-dilation	1	1.61	0	0.00	-	-	-
Reduced visual acuity (RVA)	Yes	32	51.61	12	66.67	0.53	0.18-1.60	0.258
	No	30	48.39	6	33.33			
Regular garbage collection	Yes	59	95.16	18	100.00	-	-	0.341
	No	3	4.84	0	0.00			
Sex	Female	33	53.23	9	50.00	1.14	0.40-3.25	0.809
	Male	29	46.77	9	50.00			
Source of water for the garden.	Treated water	27	43.55	8	44.44	0.96	0.33-2.77	0.946
	Stream/River	1	1.61	0	0.00	-	-	0.588
	Rain	5	8.06	0	0.00	-	-	0.213
	Well/Reservoir	8	12.90	1	5.56	2.52	0.29-21.60	0.385
Spherical equivalente	Normal vision	14	22.58	3	16.67	-	-	-
	Myopia	23	37.10	2	11.11	2.46	0.36-16.62	0.644
	Hyperopia	25	40.32	13	72.22	0.41	0.10-0.70	0.353
Swims in the river, lagoon, or pond	Yes	1	1.61	0	0.00	-	-	0.588
	No	61	98.39	18	100.00			
Vascular narrowing	Present	0	0.00	62	100.00	-	-	-
	Present	0	0.00	18	100.00			
Vasculitis	Present	0	0.00	62	100.00	-	-	-
	Absent	0	0.00	18	100.00			
Vitritis	Present	0	0.00	0	0.00	-	-	-
	Absent	62	100.0	18	100.00			
There are cats in the neighborhood	Yes	56	90.32	16	88.89	1.17	0.21-6.35	0.858
	No	6	9.68	2	11.11			
There are dogs in the neighborhood	Yes	55	88.71	16	94.12	0.49	0.06-4.29	0.513
	No	7	11.29	1	5.88			

Walks barefoot	Yes	15	24.19	4	22.22	1.12	0.32-3.91	0.863
	No	47	75.81	14	77.78			
Water source	Piped	45	72.58	8	44.44	0.47	0.16-1.40	0.170
	Well	17	27.42	10	55.56			
Which animal(s) do you have?	None	26	41.94	9	50.00	0.72	0.25-2.07	0.544
	Horse	2	3.23	3	16.67	0.17	0.02-1.09	0.038
	Rabbit	0	0.00	0	0.00	-	-	-
	Chicken	29	46.77	6	33.33	1.76	0.58-5.28	0.311
	Bird	2	3.23	1	5.56	0.57	0.05-6.63	0.647
	Pig	5	8.06	3	16.67	0.44	0.09-2.05	0.284
	Pigeon	0	0.00	0	0.00	-	-	-
	Cow	5	8.06	4	22.22	0.31	0.07-1.29	0.094

n: number, %: percentage, OR: Odds Ratio, CI: Confidence Interval, *p*: Significance Level

Table S3. Sociodemographic characteristics, analysis of epidemiological and ocular characteristics related to seropositivity for *Toxoplasma gondii* in children and elderly individuals

Sociodemographic, epidemiological and ocular health factors		n (Positive)/ Total		n (Negative)/ Total		OR	CI95%	*p*
		n.	%	n.	%			
Age	≥ 5 ≤ 7	38	38.00	43	70.49	4.07	2.05-8.06	0.00004
	≥ 60	62	62.00	18	29.51			
Active chorioretinitis presence	Yes	0	0.00	0	0.00	-	-	-
	No	100	100.0	61	100.00			
Anterior chamber and iris	Altered	0	0.00	0	0.00	-	-	-
	Normal	100	100.0	61	100.00			
Astigmatism	Yes	37	37.00	21	34.43	1.12	0.57-2.18	0.741
	No	63	63.00	40	65.57			
Bathroom with toilet bowl	Yes	93	93.00	58	95.08	0.69	0.17-2.76	0.595
	No	7	7.00	3	4.92			
Chorioretinitis scar lesion	Yes	3	3.06	1	1.64	1.89	0.19-18.94	0.971
	No	95	96.94	60	98.36			
Cornea	Altered	6	5.00	0	0.00	-	-	-
	Normal	94	94.00	61	100.00			
Distance visual acuity (DVA)	20/20	34	34.00	31	50.82	-	-	-
	20/30 - 20/60	21	21.00	19	31.15	1.01	0.46-2.22	0.458
	20/70 - 20/160	20	20.00	7	11.48	2.60	0.97-7.00	0.089
	20/200 - 20/400	8	8.00	2	3.28	3.65	0.72-15.50	0.193
	Blindness (CD)	12	12.00	1	1.64	10.94	1.34-89.10	0.018
	SPL	4	4.00	0	0.00	-	-	-
	PL	0	0.00	1	1.64	-	-	-
Drinks water from a well, pond, or river	Yes	33	33.00	22	36.07	0.87	0.45-1.72	0.691
	No	67	67.00	39	63.93			
Eats raw or undercooked meat	Yes	18	18.00	82	18.03	0.99	0.44-2.28	0.996
	No	11	82.00	50	81.97			
Eye deviation	Yes	1	1.00	0	0.00	-	-	0.433
	No	99	99.00	61	100.00			
Goes to a farm, ranch, or poultry farm	Yes	51	51.00	41	67.21	**0.51**	**0.26-0.98**	**0.044**
	No	49	49.00	20	32.79			
Goes to the beach	Yes	34	34.00	23	37.70	0.85	0.44-1.65	0.633

	No	66	66.00	38	62.30			
Has a Garden	Yes	65	65.00	20	32.79	**3.80**	**1.94-7.47**	**0.0001**
	No	35	35.00	41	66.21			
Has a yard with soil	Yes	95	95.00	53	95.08	2.87	0.89-9.21	0.145
	No	5	5.00	8	4.92			
Has any illness	Yes	38	38.00	10	16.39	**3.13**	**1.42-6.88**	**0.004**
	No	62	62.00	51	83.61			
Has cats	Yes	63	63.00	15	28.81	**5.56**	**2.74-22.27**	**0.0001**
	No	37	37.00	49	76.19			
Has dogs	Yes	58	58.00	41	67.21	0.67	0.35-1.31	0.244
	No	42	42.00	20	32.79			
Has had parasitic infections.	Yes	68	68.00	34	55.74	1.69	0.87-3.26	0.117
	No	32	32.00	27	44.26			
Has noticed any vision problems	Yes	63	63.00	23	37.70	2.81	1.46-5.43	0.002
	No	37	37.00	38	62.30			
Household waste disposal	Septic tank	90	90.00	59	96.72	0.30	0.06- 1.44	0.115
	Open air	10	10.00	2	3.28			
Lens	Opaque	37	39.78	10	16.67	3.30	1.49-7.32	0.002
	Normal	56	60.22	50	83.33			
Maternal ethnicity	mixed race	39	39.00	29	47.54	0.70	0.37-1.34	0.368
	Black	61	61.00	32	52.46			
Ocular itching	Yes	7	7.00	2	3.28	2.22	0.45-11.05	0.319
	No	93	93.00	59	96.72			
Ocular motility	Normal	99	99.00	61	100.00	-	-	0.433
	Permanent exotropia (XT)	1	1.00	0	0.00			
Ocular redness	Yes	8	8.00	3	4.92	1.68	0.43-6.60	0.452
	No	92	92.00	58	95.08			
Other illnesses	Arthritis	2	2.00	0	0.00	-	-	-
	Depression	2	2.00	0	0.00	-	-	-
	Diabetes	11	11.00	4	6.56	1.76	0.53-5.80	0.347
	Dyslipidemia	7	7.00	4	6.56	1.07	0.30-3.8	0.914
	Gastritis	3	3.00	0	0.00	-	-	-
	Arterial hypertension	23	23.00	11	18.03	1.36	0.61-3.03	0.454
	Renal insufficiency	2	2.00	0	0.00	-	-	-

Parents' education level	≤ 8 years	80	80.00	35	52.46	2.97	1.46-6.02	0.004
	> 8 years	20	20.00	26	47.54			
Participant's or guardian's education level (for children)	≤ 8 years	90	90.00	57	93.44	1.58	0.47-5.29	0.452
	> 8 years	10	10.00	4	6.56			
Plays in the soil	Yes	68	68.00	28	78.69	**2.50**	**1.30-4.82**	**0.009**
	No	32	32.00	33	21.31			
Pupil	Normal	96	96.00	61	100.00	-	-	-
	Relative Afferent Pupillary Defect (RAPD)	2	2.00	0	0.00	-	-	-
	Paralytic mydriasis	1	1.00	0	0.00	-	-	-
	Paralyzed at mid-dilation	1	1.00	0	0.00	-	-	-
Reduced visual acuity (RVA)	Yes	32	32.00	12	19.67	1.92	0.90-4.10	0.089
	No	68	68.00	49	80.33			
Regular garbage collection	Yes	84	84.00	45	73.77	1.87	0.85-4.08	0.115
	No	16	16.00	16	26.23			
Sex	Female	52	52.00	29	47.54	1.19	0.63-2.26	0.583
	Male	48	48.00	32	52.46			
Source of water for the garden	Treated water	36	55.38	15	62.50	0.74	0.28-1.95	0.547
	Stream/River	4	6.15	0	0.00	-	-	0.056
	Rain	5	7.69	0	0.00	-	-	0.216
	Well/Reservoir	21	32.31	9	37.50	0.79	0.30-2.11	0.646
Spherical equivalente	Normal vision	42	42.00	35	57.38	-	-	-
	Myopia	31	31.00	8	13.11	3.23	1.32-7.92	0.015
	Hyperopia	27	27.00	18	29.51	1.25	0.59-2.64	0.691
Swims in the river, lagoon, or pond	Yes	70	70.00	45	73.77	0.83	0.41-1.69	0.607
	No	30	30.00	16	26.23			
Vascular narrowing	Present	0	0.00	0	0.00	-	-	-
	Present	100	100.0	61	100.00			
Vasculitis	Present	0	0.00	0	0.00	-	-	-
	Absent	100	100.0	61	100.00			
Vitritis	Present	0	0.00	0	0.00	-	-	-
	Absent	100	100.0	61	100.00			
There are cats in the neighborhood	Yes	86	86.00	46	75.41	2.00	0.89-4.51	0.090
	No	14	14.00	15	24.59			

There are dogs in the neighborhood	Yes	85	85.00	53	86.89	0.85	0.34-2.15	0.740
	No	15	15.00	8	13.11			
Walks barefoot	Yes	43	43.00	33	54.10	0.64	0.34-1.21	0.171
	No	57	57.00	28	45.90			
Water source	Piped	89	89.00	50	81.97	0.56	0.23- 1.39	0.207
	Well	11	11.00	11	18.03			
Which animal(s) do you have?	None	63	63.00	41	67.21	-	-	-
	Horse	11	11.00	9	14.75	1.26	0.47-3.29	0.828
	Rabbit	2	2.00	0	0.00	-	-	-
	Chicken	51	51.00	35	57.38	0.83	0.59-1.89	0.976
	Bird	4	4.00	3	4.92	0.59	0.24-5.42	0.826
	Pig	10	10.00	7	11.48	0.63	0.38-3.05	0.896
	Pigeon	0	0.00	0	0.00	-	-	-
	Cow	12	12.00	12	19.67	0.24	0.63-3.75	0.472

n: number, %: percentage, OR: Odds Ratio, CI: Confidence Interval, *p*: Significance Level
